# Reconstruction Using Hinged Knee Replacement of an Unusual Knee Deformity as a Sequelae of Paediatric Septic Arthritis: A Case Report

**DOI:** 10.7759/cureus.82347

**Published:** 2025-04-16

**Authors:** Zakareya Gamie, Ziad Mahmoud, Nicholas Cooke

**Affiliations:** 1 Trauma and Orthopaedic Surgery, Newcastle University, Newcastle upon Tyne, GBR; 2 Trauma and Orthopaedic Surgery, University Hospital of North Tees, Stockton-on-Tees, GBR

**Keywords:** arthritis, deformity, hinged, knee, osteoarticular, reconstruction, replacement, septic, surgery

## Abstract

Patients with osteoarticular infections may have initial complications secondary to bacteraemia and late complications related to bone alterations due to infection. We present a case of a 28-year-old man with a complex deformity of his left knee as a sequelae of septic arthritis, varus with concave deformity of the lateral tibial plateau, and bony deficiency in the medial compartment. We describe how a hinged knee replacement was undertaken to deal with an unusual deformity and improve patient symptoms and function.

## Introduction

Osteoarticular infections (OAIs) are frequent in paediatrics, with an annual incidence in developed countries between 10 and 25 per 100,000 children [1]. Children below the age of two years are the most affected [2]. Most of these infections occur due to hematogenous spread during episodes of transient bacteraemia in patients who may have been asymptomatic until then. In case of osteomyelitis, the infection usually begins in the highly vascularized metaphyseal region and may spread to other regions, including adjacent joints [3]. Patients with OAI may have initial complications secondary to bacteraemia and late complications related to bone alterations due to infection. In an early report by Strong et al., 50 septic knees in patients younger than two years old at diagnosis were examined. Follow-up ranged from 1.1 to 21.3 years (average = 6.2). Twenty-four knees were deformed in varus or valgus of 5-40°. Deformity was present within 10 months of infection and thereafter was stable. It was due to displacement of the epiphysis into a metaphyseal defect, loss of epiphyseal cartilage, or both. Growth did not compensate for deformity [4]. In addition to deformity, other types of sequelae include avascular necrosis, contracture, early arthritis, and osteomyelitis [5]. Extensive management and reconstructive options have been described to deal with the deformity and contractures, which include the use of osteotomies, external fixation, and arthrodesis [5]. Knee arthroplasty cases pertaining to these complex deformities and the associated complications, however, are limited. We present a case of such deformity managed successfully with hinged knee replacement and no early complications.

## Case presentation

A 28-year-old man was referred to us on 05/03/2021 with a longstanding history of left knee symptoms. Informed consent was obtained from the patient to write the case report. This began as a child following a suspected reaction to an injection when he was a baby. This consequently resulted in him having osteomyelitis around the knee, diagnosed in December 1992, and ongoing knee stiffness and limb deformity. The symptoms of pain and stiffness have existed since childhood. He has never been pain free or had a good range of movement. As a child growing up, he had tibia and fibula fractures in October 1999 and a femoral fracture in May 2000 on the same leg, which were treated nonoperatively. He has always had a stiff knee and has been limited in his mobility, relying on supports, but has managed to maintain normal function and worked as a chef and running a pub, though he was off work due to COVID-19. He was otherwise well but had a history of ulcerations around the feet on both legs. The vascular surgeons in December 2017 diagnosed him with calf muscle failure secondary to this complex knee deformity. Over the course of the year before presentation to our department, he developed increasing stiffness in the knee and pain on mobilizing, which was significantly affecting his quality of life and limited his ability to drive.

On examination, he was a tall, thin gentleman, 6 feet and 6 inches tall, with no clear evidence of hypermobility. He walked with a stiff antalgic gait. On lying down, his left leg was clearly shorter than the right by 1-2 inches; it was stiff, he was able to maintain a straight leg raise, and was able to fully extend the knee with difficulty. He was able to flex the knee to around 30 degrees with significant crepitus. He finds that as the knee is getting stiff and he is not able to flex it, it is causing him to trip. The ligaments felt stable, but it was difficult to assess the cruciate ligaments due to the stiffness in the knee. He was fitted for a shoe raise, which helped him with his back pain. There were palpable pedal pulses with triphasic flow on handheld Doppler. The Oxford Knee Score (OKS) was 14.

**Figure 1:**
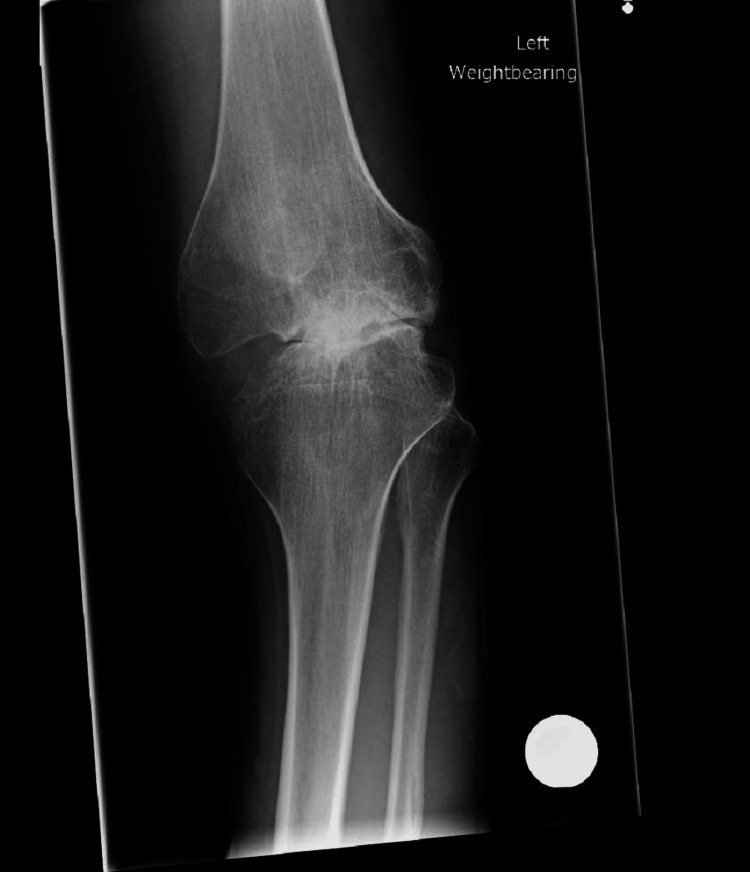
Preoperative anteroposterior X-ray of the left knee showing varus deformity and caudal sloping of the medial tibial plateau.

**Figure 2:**
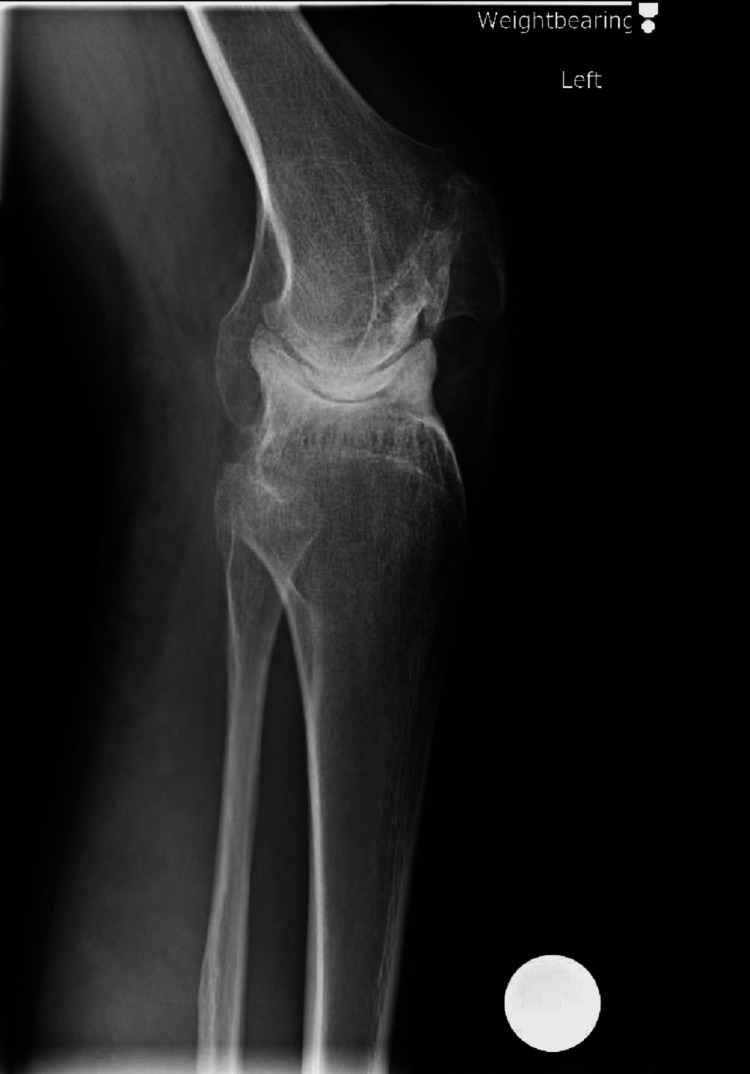
Preoperative lateral X-ray of the left knee showing concave deformity of the lateral tibial plateau.

**Figure 3:**
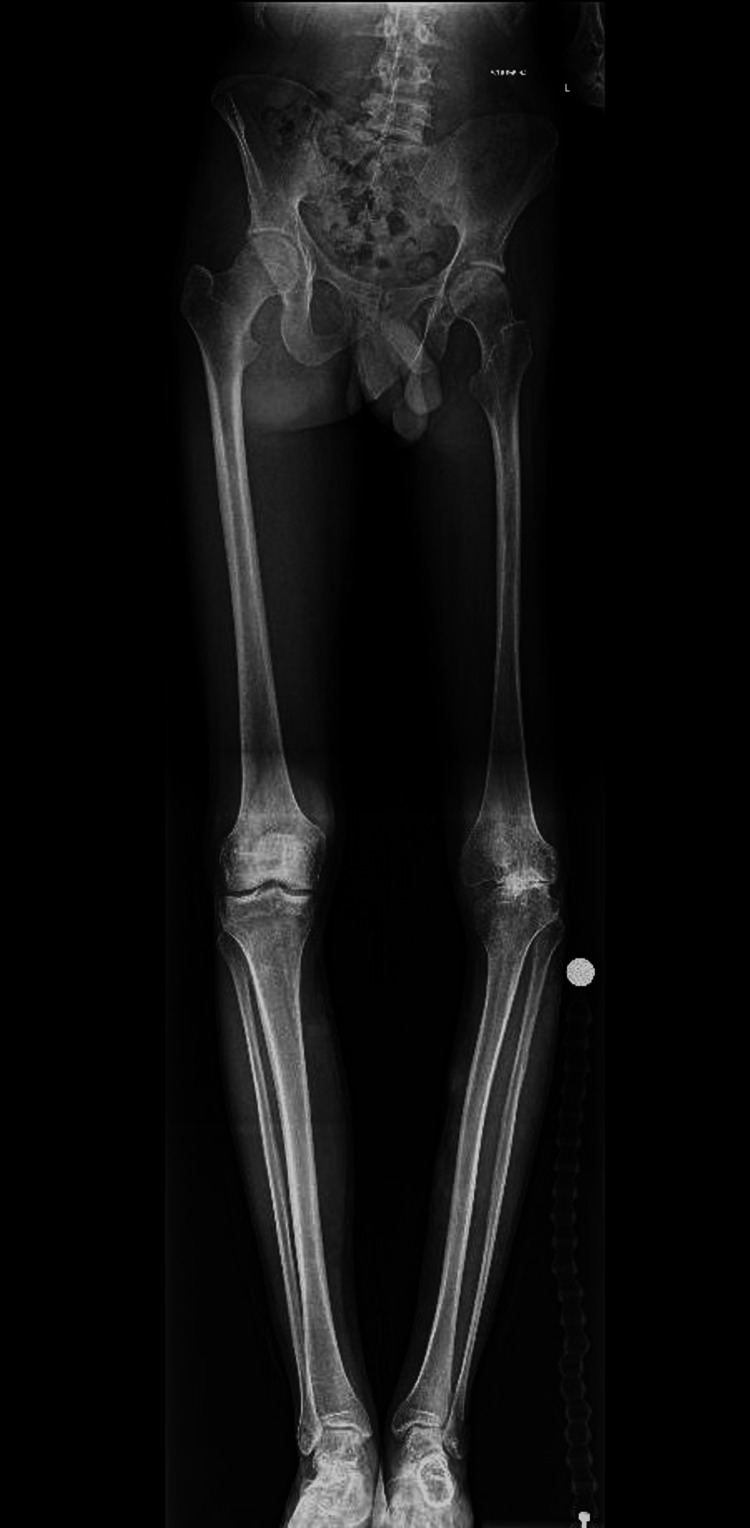
Long-leg X-ray demonstrating shortening of the left lower limb, varus deformity, and resulting hip tilt.

**Figure 4:**
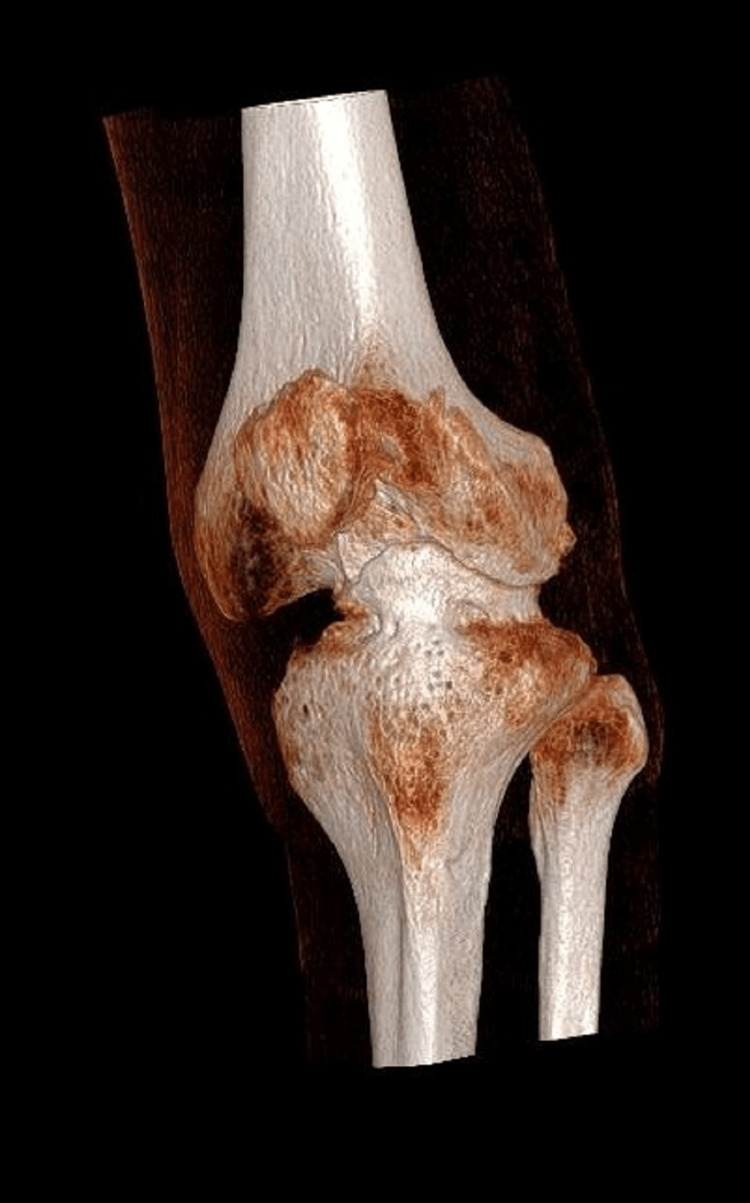
Preoperative CT reconstruction of the left knee. Significant bone loss is seen on the tibial plateau and the femoral condyles.

Left knee X-rays revealed a complex knee deformity with concave deformity of the lateral tibial plateau and bony deficiency in the medial compartment (Figures 1, 2). Long leg alignment views were requested to assess the size of the whole bone and plan surgery (Figure 3). 3D imaging was organized in the form of computed tomography (CT) and magnetic resonance imaging (MRI). CT was done to assess for knee bony deformity, follow previous septic arthritis for surgical planning, and review bone stock and alignment. X-rays and the CT scan demonstrated that he was weight bearing through the lateral aspect of the knee and had a deficient medial side (Figure 4).

**Figure 5:**
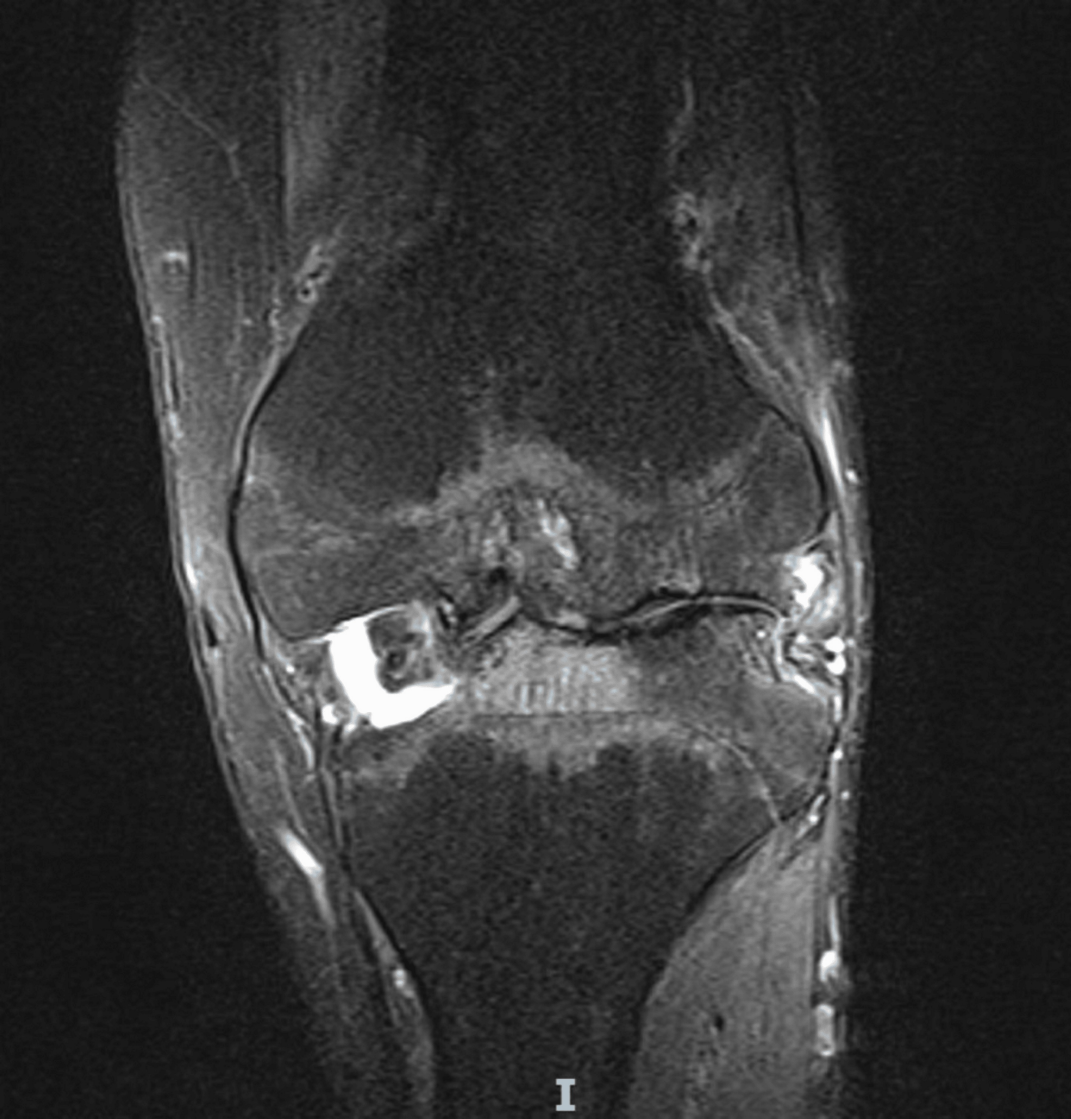
Preoperative coronal MRI of the left knee.

**Figure 6:**
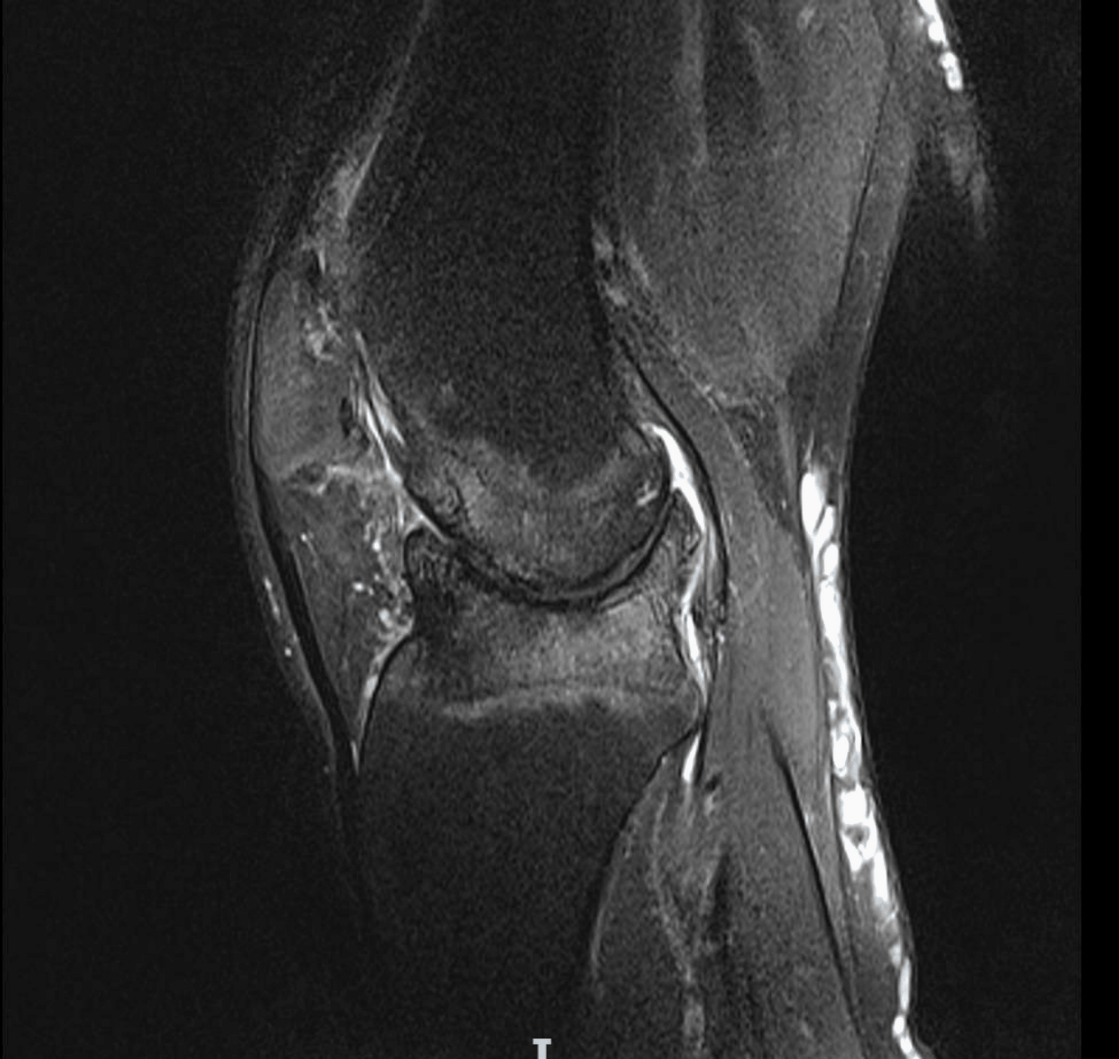
Preoperative sagittal MRI of the left knee.

MRI was undertaken due to the complex nature of the limb deformity and previous knee osteomyelitis, particularly to review the soft tissue and ligamentous integrity. It suggested that the articular deformity likely resulted from the osteomyelitis as a child, and that the collateral ligaments were intact. This was also in keeping with the clinical findings, but there was significant bone loss both on the tibial plateau and the femoral condyles. A review of the prior examinations suggested the deformity in the knee joint was the end result of osteomyelitis in infancy. The main abnormality was bony deformity/dysplasia of the femoral condyles, including the trochlear notch. There was an osteochondritis dissecans-like defect in the medial femoral condyle with a 13 mm loose body. There was a concave deformity of the lateral tibial plateau and caudal sloping of the medial tibial plateau. Complete loss of articular cartilage in all compartments was noted, with subchondral reactive bone oedema and cysts. The menisci were absent. The collateral ligaments were intact. The cruciate ligaments were hypoplastic but probably intact; however, it was difficult to be certain (Figures 5, 6).

Any form of intervention was expected to require a significant number of augmentations in terms of reconstructing an appropriate knee joint, which is likely to require stems and possible additional constraints such as a hinge. Surgical solutions would be complex, and there would be a high future risk of failure, and ultimately, the end-stage procedure could be amputation. The patient fully consented to management options, operative vs. nonoperative. Further to this, he opted for replacement over fusion, given his young age and wanting movement (also expected hip/ankle pain with fusion), but was also aware of possible complications of having a complex knee at such a young age.

Blood tests on 17th January 2022 revealed normal inflammatory markers: white blood cells = 7.7, C-reactive protein = 1.7, and erythrocyte sedimentation rate = 2 (Table 1). Bone biochemistry conducted in August 2020 was normal (Table 2), and the decision was made to proceed to a complex left total hinged knee replacement.

**Table 1:** Laboratory findings of the patient with the normal range for comparison.

Parameter	Value	Normal range	Unit
White blood cells	7.7	4.0 – 10.0	x10^9/L
Haemoglobin	145	130 – 180	g/L
Platelets	268	150 – 450	X10^9/L
Mean corpuscular volume	88.4	77 – 101	fl
Red blood cells	4.76	4.50 – 6.00	x10^12/L
Hematocrit	0.421	0.40 – 0.52	
Mean corpuscular haemoglobin	30.5	27 – 32	pg
Red cell distribution width	13.9	10.9 – 15.1	%
Neutrophils	4.6	2.0 – 7.0	x10^9/L
Lymphocytes	1.7	1.0 – 3.0	x10^9/L
Monocytes	0.7	0.2 – 1.0	x10^9/L
Eosinophils	0.5	0.0 – 0.5	x10^9/L
Basophils	0.1	0.0 – 0.1	x10^9/L
Urea	5.0	2.8 – 7.2	mmol/L
Creatinine	70	59 – 104	umol/L
Sodium	142	133 – 146	mmol/L
Potassium	4.3	3.5 – 5.3	mmol/L
Estimated glomerular filtration rate	>90	>90	ml/min/1.73m^2
C-reactive protein	1.7	0 – 5	mg/L

**Table 2:** Bone biochemistry. Normal bone biochemistry blood tests.

Parameter	Value	Normal range
Alkaline phosphatase	78 U/L	30 – 120
Albumin	44 g/L	35 – 50
Calcium (total)	2.39 mmol/L	2.20 – 2.60
Adjusted calcium	2.37 mmol/L	2.20 – 2.60
Phosphate	1.09 mmol/L	0.8 – 1.5

Operative procedure

Reconstruction of the patient's knee using a hinged knee replacement with augments and hinges was considered, as there was ligamentous instability in all directions. It took place on 14th September 2022. The operation was carried out under general anaesthesia. A tourniquet at 300 mmHg was used for 60 minutes. Cefuroxime 1.5g IV was given at induction. Preoperative range of movement was 20-40°. Varus deformity with articulation was noted mainly laterally. A midline incision with medial parapatellar approach was used. Significant medial and lateral collateral ligament laxity was noted. The femur was 6° intramedullary, with standard distal resection of +2 mm, and the femur was found to be internally rotated. The tibia was 0° extramedullary, with 10 mm lateral resection and therefore required a 10 mm medial augment (tibial block size 4 with two screws). The tibial canal was reamed to 10 mm, and a size 3 tibial tray with stem was used. Due to femur internal rotation, the posterior condyle resection was cut to be parallel with the tibia. The measure was size D with a stem, and a 5 mm posterior femoral lateral augment was used. The patella was resurfaced, and it was found to be very small (size = 26 mm). Full extension was achieved, with flexion to 70° when the components were trialled. The components inserted and cemented were as follows: NexGen® Complete Knee Solution (Zimmer®, Warsaw, IN), rotating hinge knee with hinge post extension, and size D femur with 12 mm height. Stem extension was 13 x 100 mm (combined length = 145 mm). Tibial component size was 3 with stem extension of 15 x 30 mm (combined length = 75 mm). The components were cemented using Copal G+V (Heraeus Medical GmbH, Wehrheim, Germany). All-poly patella size 26, with 7.5 mm thickness, was used. Closure was in layers using Vicryl, polydioxanone (PDS), and Monocryl. Local anaesthetic was infiltrated. The postoperative plan included two doses of cefuroxime 750 mg IV at eight and 16 hours, check X-ray and blood tests, removal of the drain, routine deep venous thrombosis (DVT) prophylaxis, and mobilization.

Outcome

**Figure 7:**
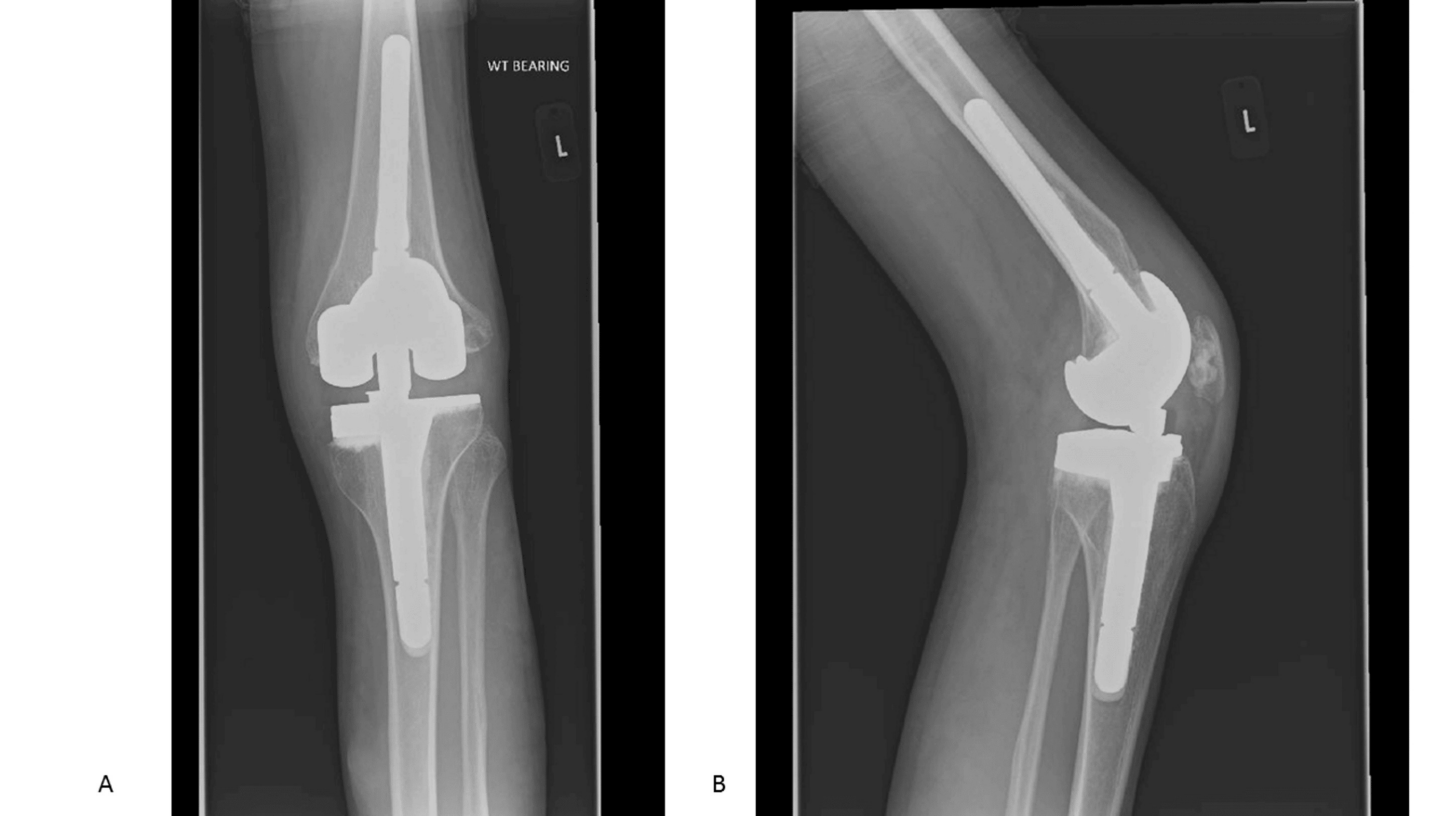
(A) Immediate postoperative anteroposterior X-ray of the left knee. (B) Immediate postoperative lateral X-ray of the left knee.

**Figure 8:**
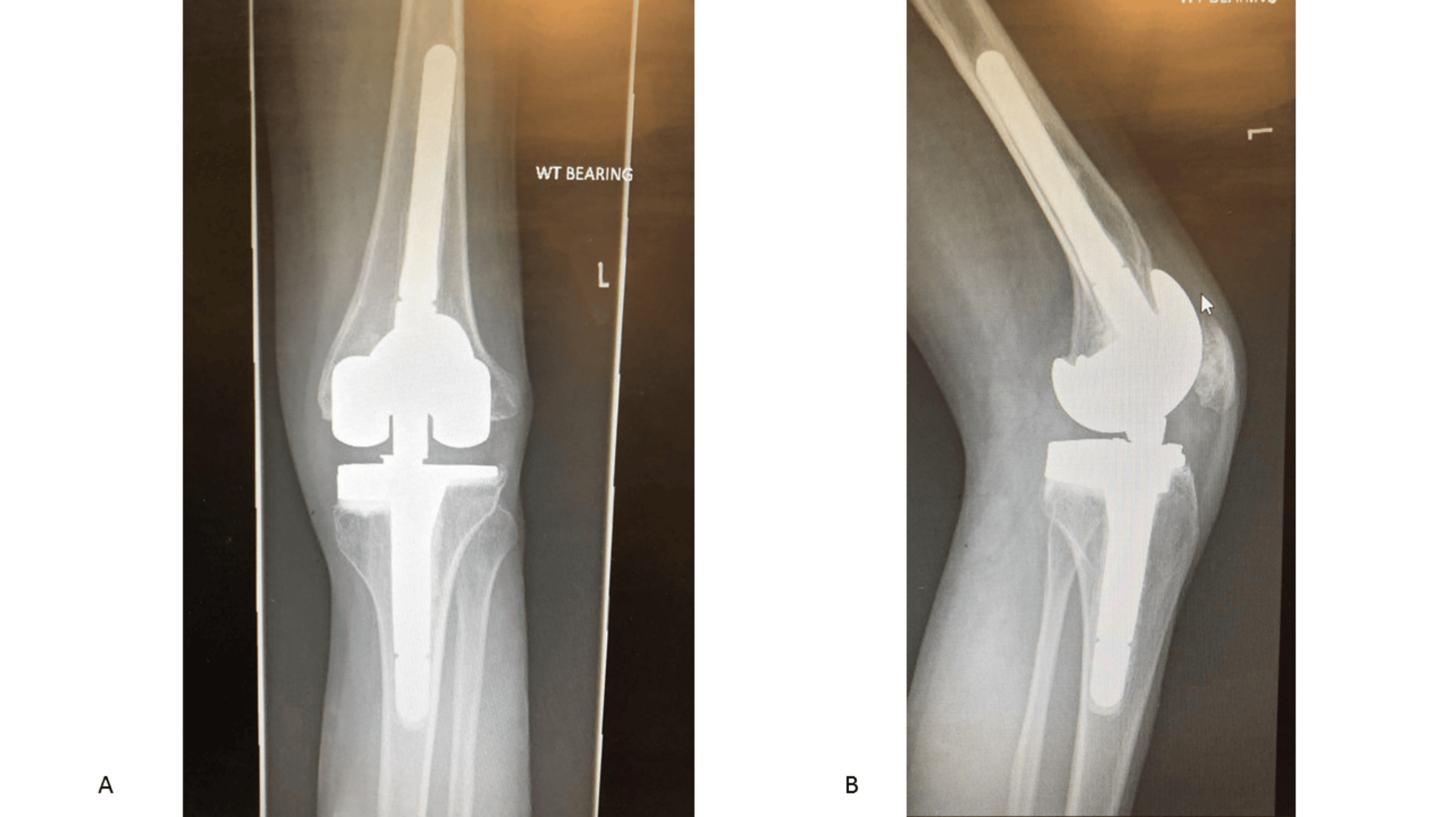
(A) Postoperative anteroposterior X-ray of the left knee at two years and seven months. (B) Postoperative lateral X-ray of the left knee at two years and seven months.

Postoperative imaging was done at day one and week 15 with good results (Figure 7). Follow-up was undertaken on 3/11/2022 (19 days postoperatively). The range of motion (ROM) was 20-30 degrees, and pain improved with no wound complications. Follow-up was also carried out on 10/1/2023 (four months postoperatively). The ROM was 5-50 degrees, pain was resolved, and mobilization was unaided. Intraoperative tissue and fluid samples were negative for infection. Follow-up at two years and seven months revealed that he was fully employed with no pain or signs of infection. His ROM was 0-45 degrees. Check X-rays (Figure 8) looked satisfactory and unchanged when compared to immediate postoperative X-rays. The OKS was 47.

## Discussion

We have described in this case report a complex knee deformity as a result of septic arthritis in childhood, treated with a hinged knee replacement, with a good outcome in terms of symptom relief and mobilization. In the literature, reports detail the management following septic arthritis in the hip as a child, with improved surgical techniques to enable management of deformities in young and active patients. However, the incidence of complications such as fractures, dislocation, nerve injury, and recurrence of infection in this group of patients can be high [6].

The role of arthroplasty in the management of septic arthritis remains to be clearly defined. Joint replacement provides a much better functional result than arthrodesis or resection arthroplasty, but incurs a risk of septic failure, which is difficult to assess [7]. Moreover, in case of previous joint infection quiescent for long enough to be considered to be resolved, the onset of functional joint deterioration raises the question of degenerative joint pathology or late resurgence of infection [7]. The role of arthroplasty and treatment strategy as a whole (preoperative assessment, one- or two-stage surgery, antibiotic prophylaxis, postoperative antibiotic therapy, and follow-up) in this situation of doubt is not clearly established. The body of evidence is greater for total hip arthroplasty (THA) than total knee arthroplasty (TKA), which is the most successful method for treating sequelae of suppurative arthritis of the hip [8,9]. However, THA for the treatment of the late sequelae of septic arthritis of the hip is reported to be technically demanding because of abnormal bone development, severe flexion deformities resulting from severe soft tissue contractures, the relatively young age of patients, potential risk of reinfection, and higher complication rates [8-10].

There are reports detailing performing TKA for the previously infected adult native knee; however, there is currently no standard in the treatment strategy for this group of patients [11]. As the time from diagnosis of native knee septic arthritis to total arthroplasty increases, the risk of subsequent infection decreases [11]. There is minimal literature and unclear metrics to guide surgeons on when elective arthroplasty should be performed in patients with prior septic arthritis and the management of complex deformities using arthroplasty [12,13]. The International Consensus Meeting (ICM) on Periprosthetic Joint Infection in 2013 determined that research on the optimal timing of elective arthroplasty for patients with prior septic arthritis remains one of the most important areas of future research [14]. In the largest series of patients with septic arthritis undergoing total joint arthroplasty (TJA), Kim et al. investigated 170 hips in 161 patients with previous childhood septic arthritis and concluded that there should be a quiescent period of 10 years after the infection before an arthroplasty is performed [9,15]. The current ICM recommendation stated that “in the absence of concrete evidence, we recommend that arthroplasty be delayed at least until completion of antibiotic treatment and resolution of clinical signs of infection, but no earlier than three months from the inciting event” [16].

Despite developments in antibiotics, infection diagnosis, and control, there remains a requirement for research and implementation of new technologies and greater understanding of prosthetic joint infection and recurrence of infection [17]. A case series of 53 patients with septic arthritis treated with arthroplasty concluded that management of septic arthritis by arthroplasty following the present protocol (two-stage implantation in evolutive and one-stage implantation in quiescent arthritis) gave very good functional results in both knee and hip, with 87% of eradication of infection in evolutive septic arthritis and 95% in quiescent septic arthritis. No clinical, microbiological, or treatment-related risk factors for failure emerged [18]. A retrospective case-control study on 215 primary total knee arthroplasties done for patients with history of native knee septic arthritis found that there was a 6.1-fold increased risk of prosthetic joint infection in patients undergoing TKA with a history of native knee septic arthritis when compared with controls undergoing TKA for the treatment of osteoarthritis, with a cumulative incidence of 9% at 10 years [11].

The focus of this current case report was the severity of the deformity that can occur with septic arthritis of the knee in childhood, and how this can be dealt with using the modern hinge knee prosthesis for such challenging cases that can present with malalignment, bone loss, and instability. The current case was undertaken as a one-stage procedure. There has been close monitoring for any complications such as reinfection, as it can be a critical complication, as well as osteolysis and loosening. There have been no signs clinically or radiographically of the aforementioned complications at two years and seven months.

## Conclusions

The development of surgical techniques and arthroplasty implants has enabled us to deal with significant knee deformity that can occur as a result of a history of septic arthritis in childhood. The uniqueness is the nature of the deformity that can result from an osteoarticular infection. The use of a hinged knee replacement can be utilized despite this nature and degree of deformity. Use of hinged knee replacement with augments can deal with issues such as bone loss and instability. Patients can show clinical improvement in terms of mobilization, knee function, and patient satisfaction.
